# Supplementary material for: Single-cell proteomic and transcriptomic analysis of macrophage heterogeneity using SCoPE2
Source: Genome Biol. 2021 Jan 27;22:50. doi: 10.1186/s13059-021-02267-5 (PMC7839219; doi:10.1186/s13059-021-02267-5)
Supplement: Supplementary file 1 — Additional file 1. contains Fig. S1-S10. Pdf file with all supplementary figures (Fig. S1-S10) with corresponding figure legends. [file 13059_2021_2267_MOESM1_ESM.pdf]

Additional file 1: Supplementary figures

Single-cell proteomic and transcriptomic analysis of macrophage heterogeneity using SCoPE2

Harrison Specht,[1] Edward Emmott,[1,2] Aleksandra A. Petelski,[1] R. Gray Huffman,[1] David H. Perlman,[1,3] Marco Serra,[4] Peter Kharchenko,[4] Antonius Koller,[1] Nikolai Slavov[1,✉]

[1]Department of Bioengineering and Barnett Institute, Northeastern University, Boston, MA 02115, USA
[2]Current address: Centre for Proteome Research, Department of Biochemistry, University of Liverpool, Liverpool, L69 7ZB, UK
[3]Current address: Merck Exploratory Sciences Center, Merck Sharp & Dohme Corp., 320 Bent St. Cambridge, MA 02141
[4]Department of Biomedical Informatics, Harvard Medical School, Boston, MA 02115, USA

✉ Correspondence: nslavov@alum.mit.edu
∈ Data, code & protocols: scope2.slavovlab.net

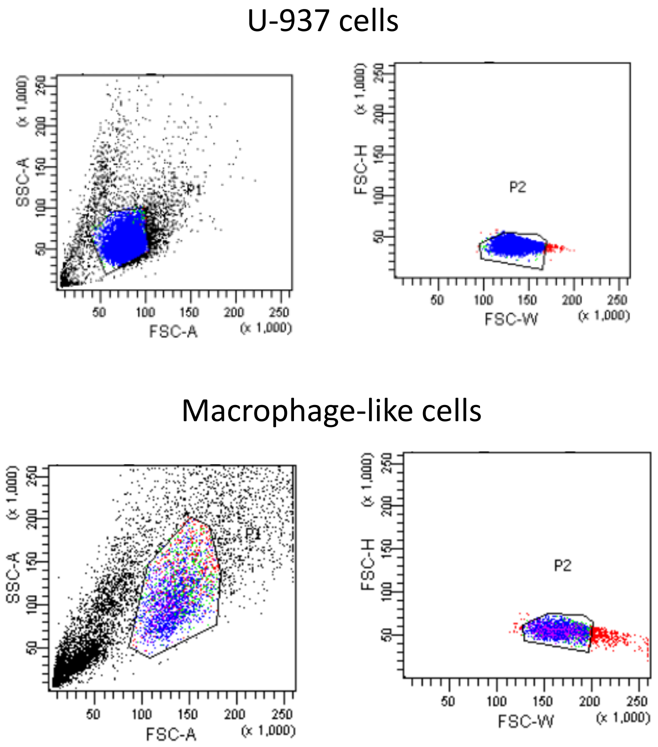

Fig. S1. FACS gating for sorting single monocyte and macrophage-like cells.
Representative FACS gatings are shown for the selection of live monocyte and macrophage-like cells, separating them from debris, dead cells, or granules. Secondary gating was used to decrease the probability of sorting doublets.

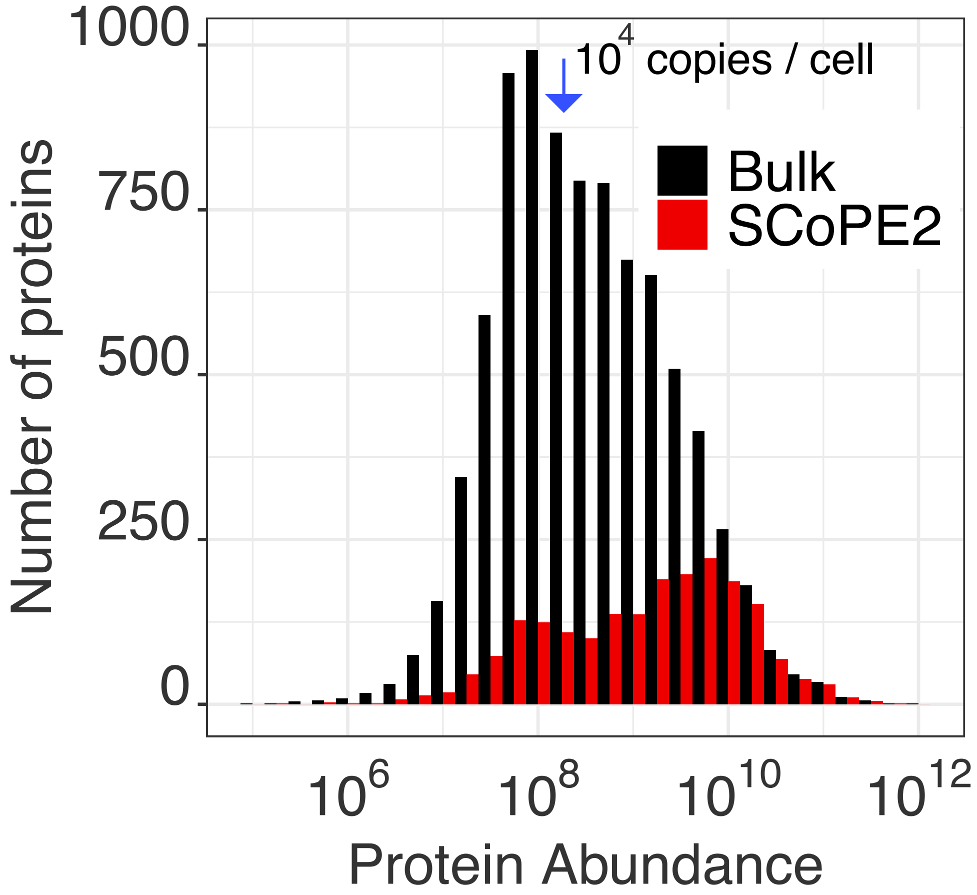

Fig. S2. Protein abundance estimates for proteins quantified by SCoPE2
Rieckmann et al. [10] analyzed a bulk sample of classical monocytes and estimated (as LFQ intensity) the abundance of about 9,500 proteins, displaced as a black distribution. The subset of these proteins that were also quantified in single cells from SCoPE2 sets are displayed as a red distribution. The median LFQ intensity is indicated with a blue arrow. Based on the protein estimate method suggested by Milo [11], the median protein copy number is roughly 10,000-20,00 protein copies per cell.

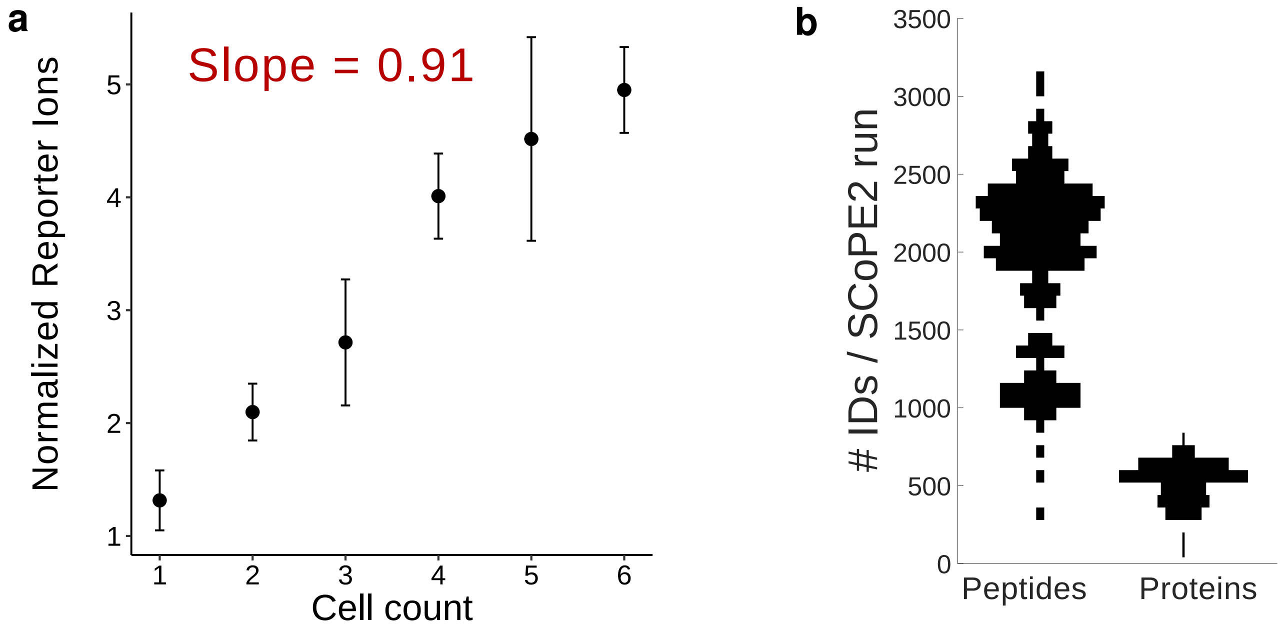

Fig. S3. Scaling of MS signal with protein input and peptide identification based on MS spectra.
(**a**) A ladder of 1 to 6 single cell equivalents. The average signal measured per SCoPE2 channel scales linearly with its input. We performed 6 experiments and varied (randomized) the TMT labels used for different input amounts. The error bars denote standard deviations between replicates. (**b**) Number of peptides and proteins identified by MaxQuant per SCoPE2 set at 1% FDR. These identifications are based only on the MS spectra and do not incorporate retention time information.

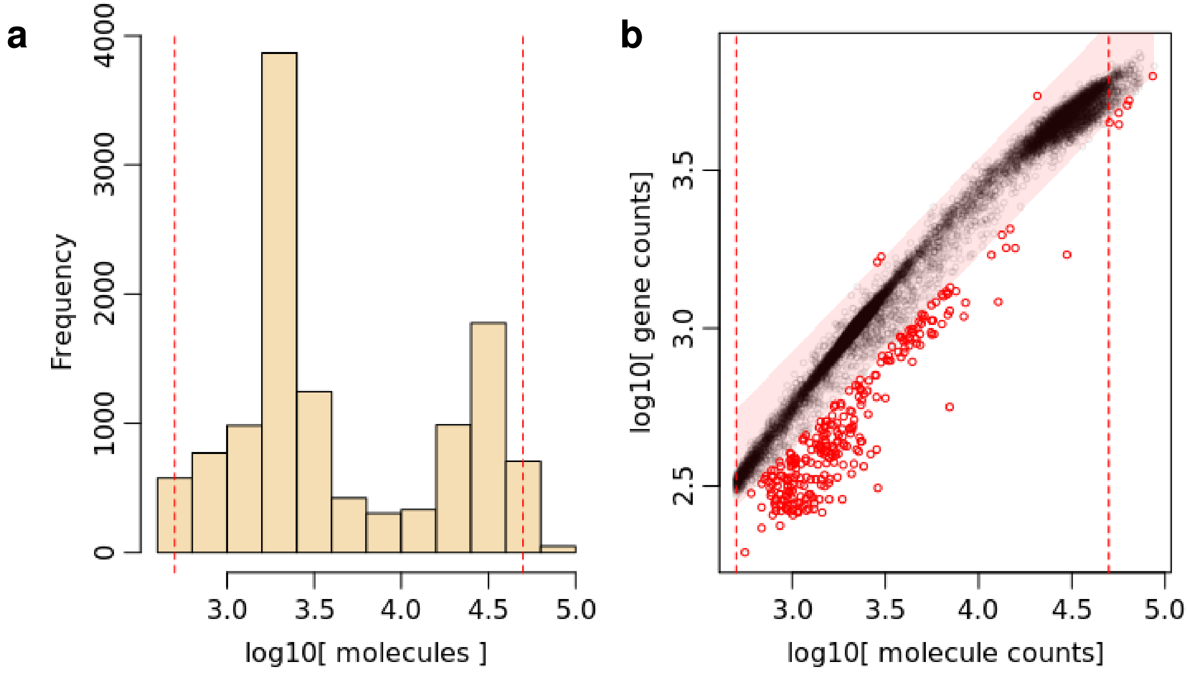

Fig. S4. Quality control plot for single-cell RNAseq data from the 10x Chromium platform.
(**a**) A distribution of number of unique molecular barcodes per cell. The left mode likely corresponds to empty droplets while the right mode corresponds to the single cells. All cells used for our analysis were sampled from the peak of the second mode. (**b**) A scatter plot of number of genes detected per cell versus the number of unique molecular barcodes per cell.

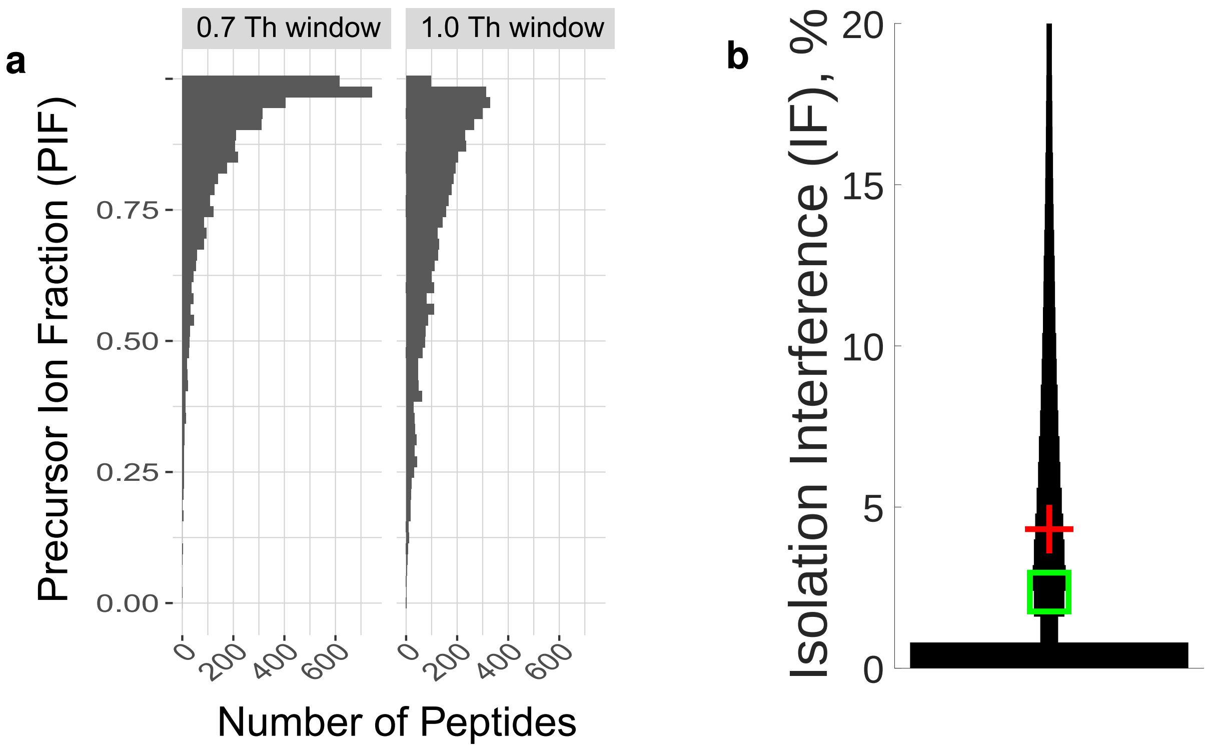

Fig. S5. Estimating the purity of MS2 spectra as a function of the width of the isolation window and by two independent search engines. (**a**) MaxQuant uses the precursor ion fraction (PIF) to estimate the fraction of the ion intensity originating from the precursor assigned to a peptide spectral match for each MS2 spectrum [4,12]. The distributions of PIF values for two controlled experiments indicate that the isolation window width used by SCoPE2 (0.7 Th) results in purer spectra compared to the isolation width (1.0 Th) used with SCoPE-MS. (**b**) Proteome Discoverer (PD) uses a completely different approach to estimate the contamination of MS2 spectra by interfering ions coisolated with the precursor ion. PD estimates Isolation Interference (IF), which should equal to $100\% - PIF$. The distribution of low IF values for the SCoPE2 data affirms the high spectral purity estimated by MaxQuant as shown in the main Figure 3d. As in main Figure 3, the green square is the median and the red plus is the mean IF.

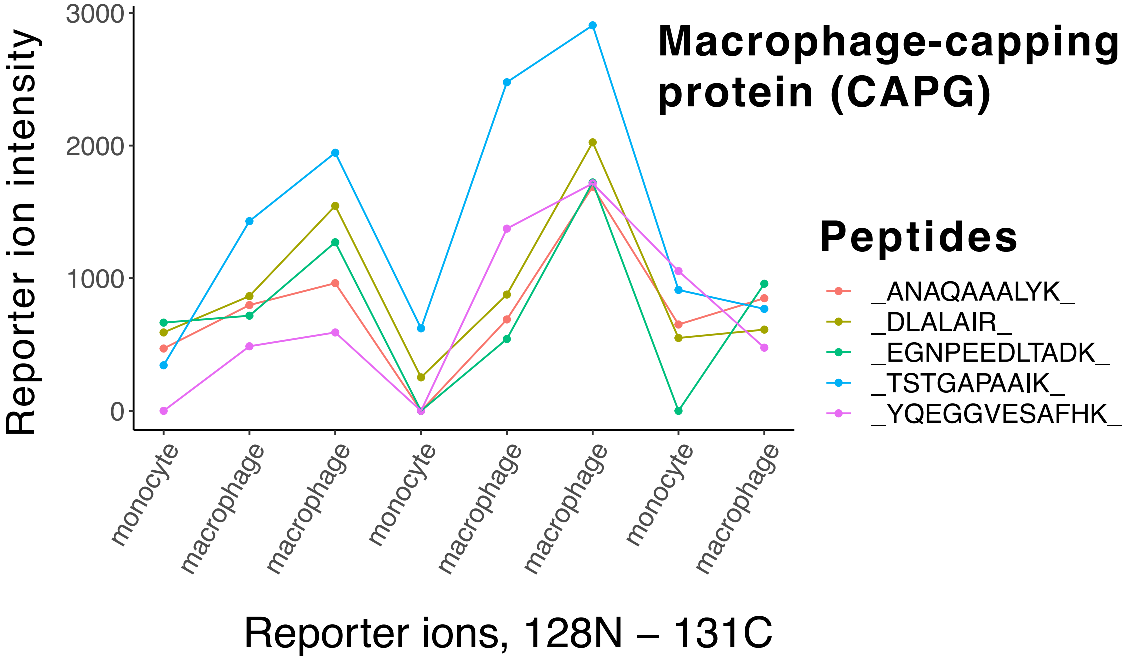

Fig. S6. Raw reporter ion intensities for macrophage-capping protein (CAPG) measured in a SCoPE2 set. The reporter ion intensities for peptides originating from CAPG in experiment 190222S-LCA9-X-FP94AP are displayed for each single cells. The sequence TSTGAPAAIKK was observed in the carrier channel, but not quantified in single cells, and thus omitted from the plot and any quantitative analysis.

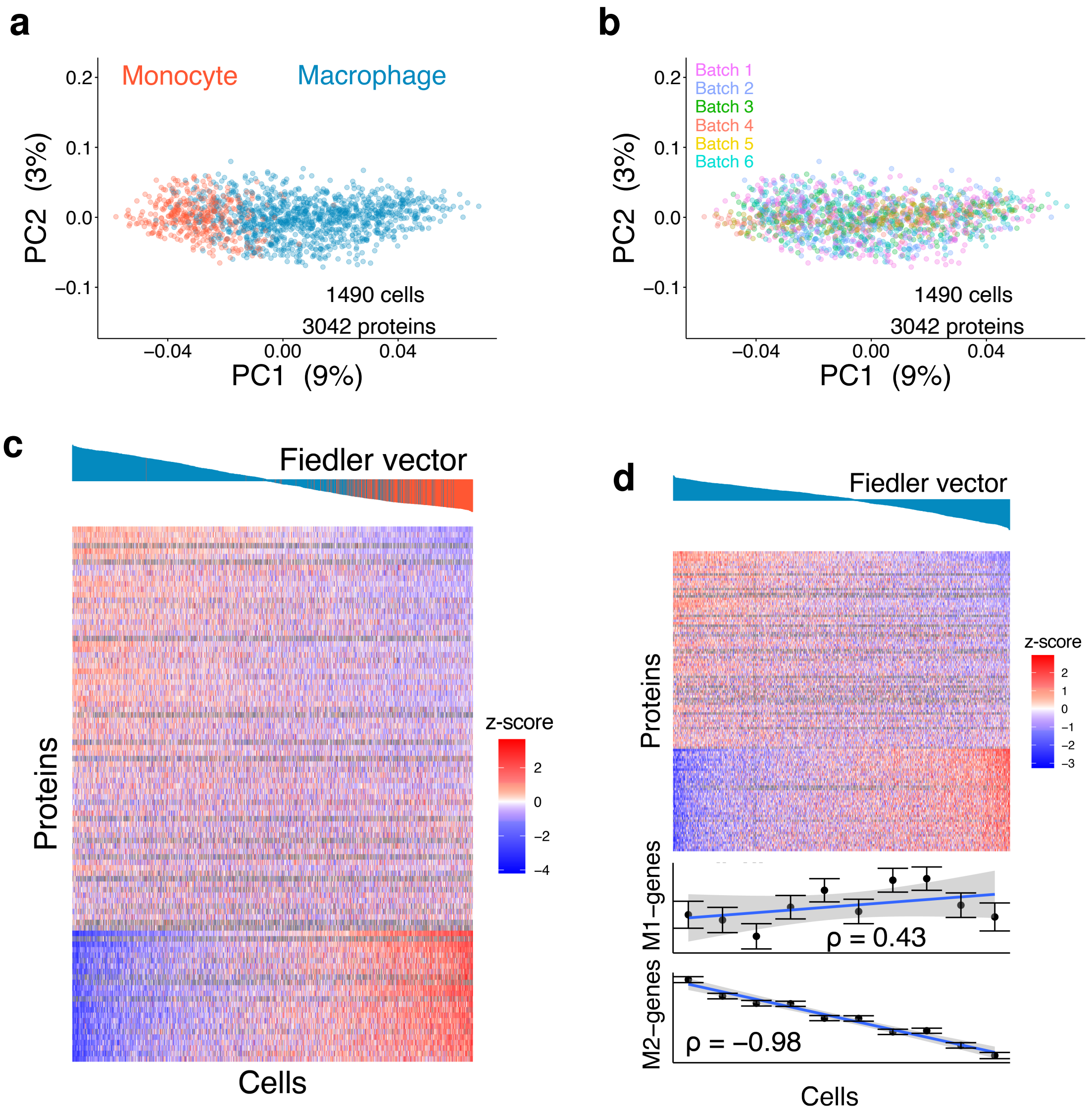

Fig. S7. Analysis without any imputation qualitatively recapitulate the results from the imputed data. (**a**) Principal component analysis was computed using only measured protein levels (without any imputation) and minimal data processing. To correct for differences between the 6 different batches of sample preparation, the levels of each protein were normalized to z-scores within each individual set by subtracting the mean and dividing by the standard deviation. The PCA analysis and data display were performed as in main Figure 4a that used imputed and batch corrected data. (**b**) Colorcoding each cell from the PCA analysis by its corresponding sample preparation batches indicates relatively uniform distribution of cells from the different batches within the 2D PCA projection space. (**c**) A heatmap from performing the spectral clustering from main Figure 5a on the raw protein measurements without imputation and batch correction. Missing data points are shown in gray. Only proteins with less than 50% missing data are displayed. (**d**) A heatmap from performing the spectral clustering from main Figure 4b on the raw protein measurements without imputation and batch correction. Missing data points are shown in gray. Only proteins with less than 50% missing data are displayed.

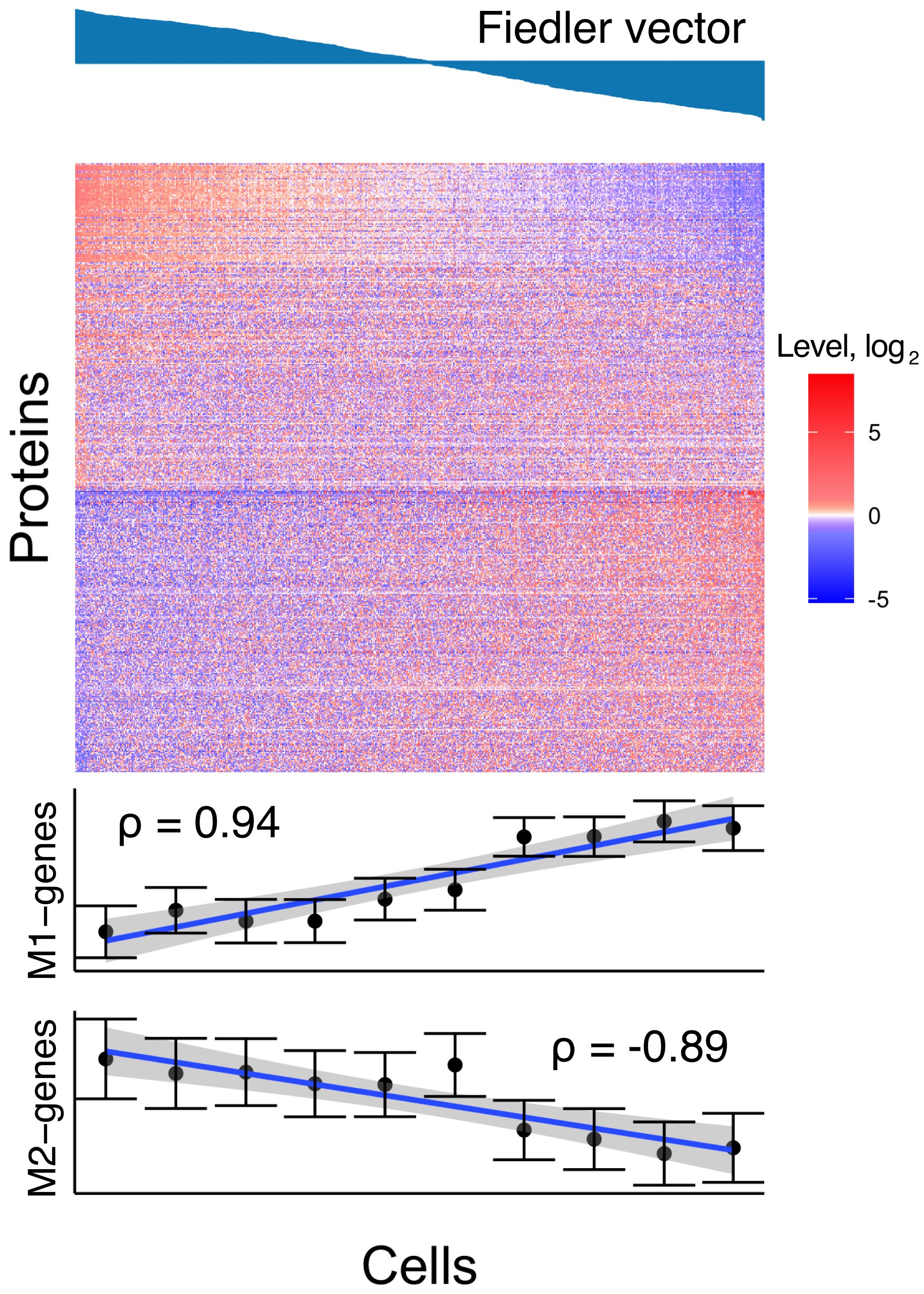

Fig. S8. Macrophage-like single cells from the mRNA data set display a gradient of polarization. The single-cell RNA data for macrophage-like cells were analyzed analogously to the protein data shown in main figure 5. Displayed are the top 25% most variable mRNA (518) levels across 425 single cells. The level for each mRNA is relative to its mean level across the 425 single cells. The levels of genes in the selected mRNA data that are enriched in M1 or M2-polarized macrophages [6] are plotted below.

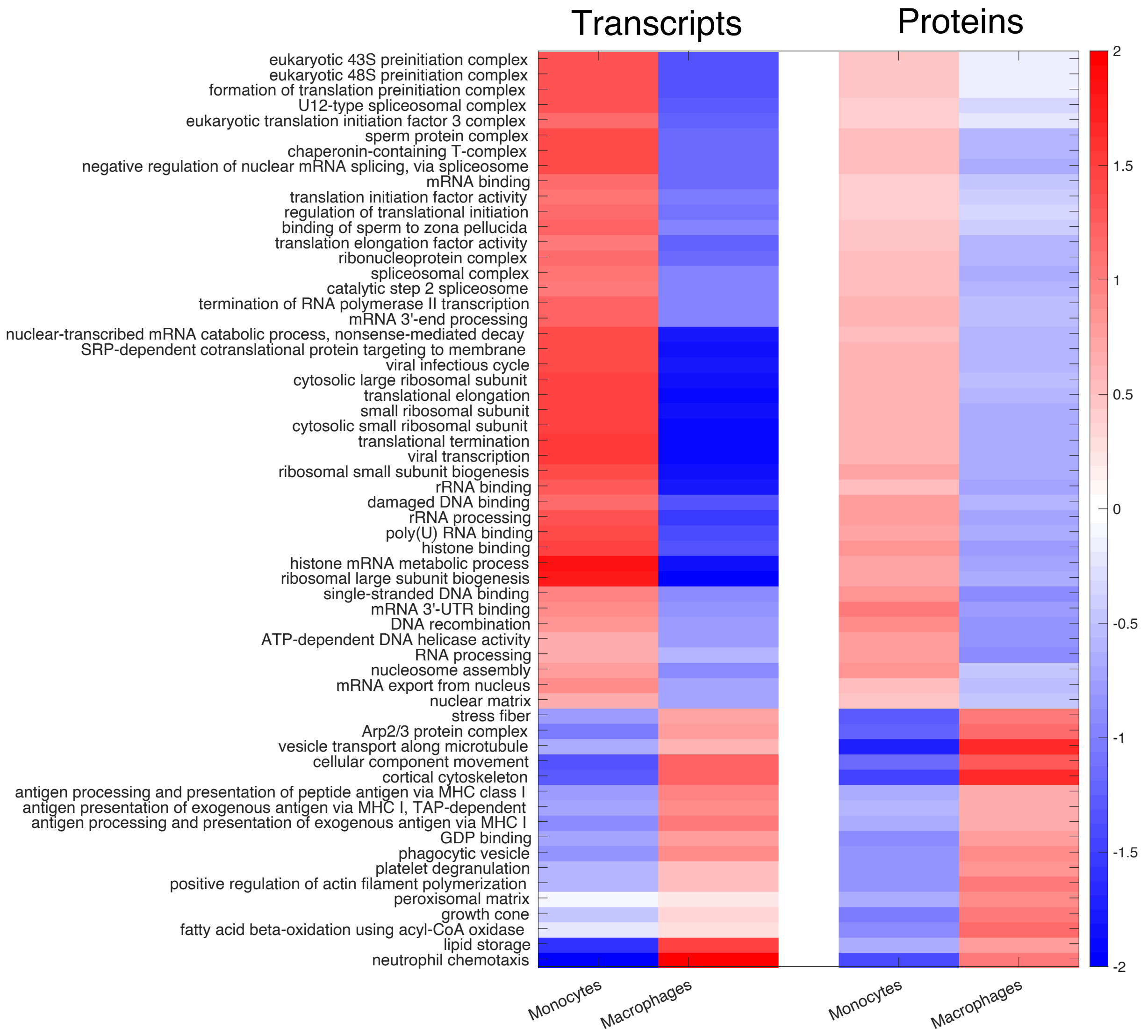

Fig. S9. Gene set enrichment analysis of cluster 1

Gene set enrichment analysis [13] identified statistically significant functional groups of genes that show differential abundance at the mRNA and protein levels in macrophages and monocytes from Cluster 1 in main Fig. 6. The colormap shows fold changes relative to the mean across all single cells on a log_2 scale.

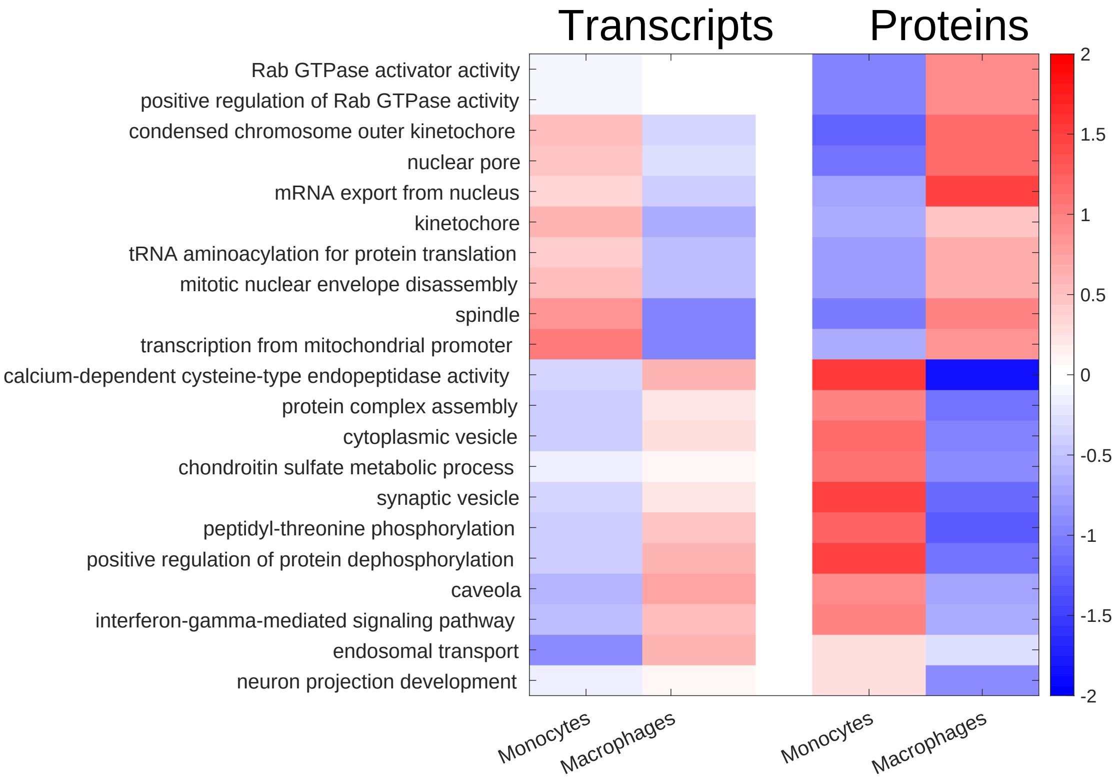

Fig. S10. Gene set enrichment analysis of cluster 2

Gene set enrichment analysis [13] identified statistically significant functional groups of genes that show differential abundance at the mRNA and protein levels in macrophages and monocytes from Cluster 2 in main Fig. 6. The colormap shows fold changes relative to the mean across all single cells on a log_2 scale.

References

1. Specht H, Harmange G, Perlman DH, Emmott E, Niziolek Z, Budnik B, et al. Minimal sample preparation for high-throughput proteomics. bioRxiv. 2019;doi:10.1101/399774.

2. Huffman G, Chen AT, Specht H, Slavov N. DO-MS: Data-Driven Optimization of Mass Spectrometry Methods. J of Proteome Res. 2019;doi:10.1021/acs.jproteome.9b00039.

3. Chen A, Franks A, Slavov N. DART-ID increases single-cell proteome coverage. PLoS Comput Biol. 2019;doi:10.1371/journal.pcbi.1007082.

4. Cox J, Mann M. MaxQuant enables high peptide identification rates, individualized ppb-range mass accuracies and proteome-wide protein quantification. Nature biotechnology. 2008;26(12):1367–1372.

5. Eden E, Navon R, Steinfeld I, Lipson D, Yakhini Z. GOrilla: a tool for discovery and visualization of enriched GO terms in ranked gene lists. BMC Bioinformatics. 2009;10(1):48.

6. Martinez FO, Gordon S, Locati M, Mantovani A. Transcriptional profiling of the human monocyte-to-macrophage differentiation and polarization: new molecules and patterns of gene expression. Journal of Immunology (Baltimore, Md: 1950). 2006;177(10):7303–7311. doi:10.4049/jimmunol.177.10.7303.

7. Budnik B, Levy E, Harmange G, Slavov N. SCoPE-MS: mass-spectrometry of single mammalian cells quantifies proteome heterogeneity during cell differentiation. Genome Biology. 2018;19:161. doi:10.1186/s13059-018-1547-5.

8. Slavov N, Dawson KA. Correlation signature of the macroscopic states of the gene regulatory network in cancer. Proceedings of the National Academy of Sciences. 2009;106(11):4079 – 4084. doi:10.1073/pnas.0810803106.

9. Barkas N, Petukhov V, Nikolaeva D, Lozinsky Y, Demharter S, Khodosevich K, et al. Joint analysis of heterogeneous single-cell RNA-seq dataset collections. Nature methods. 2019;16(8):695–698.

10. Rieckmann JC, Geiger R, Hornburg D, Wolf T, Kveler K, Jarrossay D, et al. Social network architecture of human immune cells unveiled by quantitative proteomics. Nature Immunology. 2017;18:583–593.

11. Milo R. What is the total number of protein molecules per cell volume? A call to rethink some published values. Bioessays. 2013;35(12):1050–1055.

12. Tyanova S, Temu T, Cox J. The MaxQuant computational platform for mass spectrometry-based shotgun proteomics. Nature protocols. 2016;11(12):2301.

13. Subramanian A, Tamayo P, Mootha VK, Mukherjee S, Ebert BL, Gillette MA, et al. Gene set enrichment analysis: a knowledge-based approach for interpreting genome-wide expression profiles. Proceedings of the National Academy of Sciences. 2005;102(43):15545–15550.
